# Does moonlighting influence South African nurses’ intention to leave their primary jobs?

**DOI:** 10.3402/gha.v7.25754

**Published:** 2014-12-22

**Authors:** Laetitia C. Rispel, Tobias Chirwa, Duane Blaauw

**Affiliations:** 1 Centre for Health Policy & Medical Research Council Health Policy Research Group, School of Public Health, Faculty of Health Sciences, University of the Witwatersrand, Johannesburg, South Africa; 2 Division of Epidemiology and Biostatistics, School of Public Health, Faculty of Health Sciences, University of the Witwatersrand, Johannesburg, South Africa

**Keywords:** intention to leave, turnover, moonlighting, nurses, South Africa

## Abstract

**Background:**

Staff retention and turnover have risen in prominence in the global discourse on the health workforce. Moonlighting, having a second job in addition to a primary job, has not featured in debates on turnover.

**Objective:**

This paper examines whether moonlighting is a determinant of South African nurses’ intention to leave their primary jobs.

**Design:**

During 2010, a one-stage cluster random sample of 80 hospitals was selected in four South African provinces. On the survey day, all nurses working in critical care, theatre, emergency, maternity, and general medical and surgical wards completed a self-administered questionnaire after giving informed consent. In addition to demographic information and information on moonlighting, the questionnaire obtained information on the participants’ intention to leave their primary jobs in the 12 months following the survey. A weighted analysis of the survey data was done using STATA^®^ 13.

**Results:**

Survey participants (*n*=3,784) were predominantly middle-aged with a mean age of 41.5 (SD±10.4) years. Almost one-third of survey participants (30.9%) indicated that they planned to leave their jobs within 12 months. Intention to leave was higher among the moonlighters (39.5%) compared to non-moonlighters (27.9%; *p*<0.001). Predictors of intention to leave in a multiple logistic regression were moonlighting in the preceding year, nursing category, sector of primary employment, period working at the primary job, and number of children. The odds of intention to leave was 1.40 (95% CI: 1.16–1.69) times higher for moonlighters than for non-moonlighters. The odds ratio of intention to leave was 0.53 (95% CI: 0.42–0.66) for nursing assistants compared to professional nurses and 2.09 (95% CI: 1.49–2.94) for nurses working for a commercial nursing agency compared to those working in the public sector.

**Conclusions:**

Moonlighting is a predictor of intention to leave. Both individual and organisational strategies are needed to manage moonlighting and to enhance retention among South African nurses.

In recent years, staff retention, or the extent to which health care providers remain in the health system, has risen in prominence in the global discourse on the health workforce, because of its potential to improve their availability and accessibility (1–3). The related concept of staff turnover, or the rate at which an employer loses and gains employees (4, p. 1181), has generated numerous theoretical models that provide perspectives from the fields of economics, psychology, and organisational development (5–11). Notwithstanding inconsistencies in definitions and measurement, and the lack of differentiation between voluntary (employee-initiated) and involuntary (employer-initiated) turnover (9, 12, 13), the focus of these models has mostly been on voluntary turnover (5–11).

In the health system, high turnover of skilled health professionals has both economic and non-economic consequences (2, 11, 13, 14). These include the direct and indirect costs of recruitment and staff replacement, staff shortages, increased workloads of and demands on existing staff, and the potential risks of not being able to provide safe and quality care to patients (11, 13, 14). Nurse turnover has generated an extensive body of literature over several decades (4, 11, 13–25). Much of this literature on nurse turnover focuses on high-income countries, although there is increasing attention on turnover and its predictors in low- and middle-income countries (19, 26–29). Given the numerical dominance of nurses in many countries, high turnover among nurses has the potential to affect health care provision to patients and communities (3, 11, 13, 30, 31) and the morale, performance, and productivity of the remaining nurses (13, 32).

The reasons for high nurse turnover remain complex, and may vary over time (4). Nonetheless, existing evidence suggests that the causes of high turnover include various combinations of individual, organisational, and economic factors (4, 11–13, 15). At the individual level, the literature abounds with studies that demonstrate an inverse relationship between job satisfaction in nursing and turnover (4, 5, 7, 13, 15–19, 24, 25, 30, 33, 34). An equally consistent finding is that turnover intention (or intention to leave) is the strongest antecedent of actual turnover and is also an intermediate variable between job satisfaction and turnover (7–9). However, scholars have pointed out that the notion of job satisfaction itself is multifaceted and mediated by context, the work environment, demographic characteristics, personality type, individual motives, and experiences (4, 9, 13, 17, 22, 23, 30, 35–40). At the organisational level, studies have found that the factors that influence nurse turnover include workload, work schedules, workplace stress, leadership and management styles, training and promotional opportunities and a disjuncture between nurse expectations and the reality of the workplace (7, 21, 27, 30, 36, 41–45). In terms of economic factors, existing evidence suggests that remuneration and financial rewards influence staff turnover (4, 46), but financial incentives on their own are insufficient as a retention strategy (46).

Notwithstanding the voluminous literature, there are knowledge gaps in the studies on nurse turnover. These gaps include limited empirical information on the cost of nurse turnover to a health facility or the system as a whole and the impact of turnover on patient outcomes (13). Organisational development scholars have suggested that other forms of withdrawal such as absenteeism, passive job behaviour, or moonlighting (holding a second job in addition to a primary full-time job) could precede actual turnover (8, 9, 47, 48) and deserve more attention. We could not find any empirical studies that examine the relationship between moonlighting and nurse turnover intention or actual turnover.

In South Africa, the shortages and high turnover of nurses (49) impede the implementation of major health system reforms. Moonlighting is permitted in the South African public sector under specified conditions, which includes obtaining formal permission, though not all nurses do so (50). Nonetheless, moonlighting is widespread in South Africa. A 2010 survey found that 28.0% of nurses had done moonlighting in the year preceding the survey (51). Using data from the same survey, this paper examines factors associated with intention to leave and evaluates whether moonlighting is a predictor of nurses’ intention to leave their primary jobs. The findings of the study are part of a larger research project to examine casualisation in the nursing profession.

## Methods

During 2010, a one-stage cluster random sample of 80 hospitals was selected from the four South African provinces of the Eastern Cape (predominantly rural, but with a few large cities), Free State (mixed urban and rural), Gauteng (urban), and the Western Cape (predominantly urban). The Human Research Ethics Committee (Medical) of the University of the Witwatersrand in Johannesburg provided ethics approval for the study. The relevant public and private health care authorities also provided the necessary study approvals. All participants provided written, informed consent.

In each of the four provinces, the sampling frame consisted of all public and private hospitals, stratified by type of hospital for public hospitals; and by ownership and hospital bed numbers for private hospitals. A random sample of public and private sector hospitals was then selected from each stratum proportional to the total number of hospitals in that stratum.

On the 24-hour survey day, all nurses working in critical care, theatre, emergency, maternity, and general medical and surgical wards completed a self-administered questionnaire after giving informed consent. In addition to demographic information and information on moonlighting, the questionnaire obtained information on the participants’ intention to leave their primary jobs in the 12 months following the survey. Further details of the survey methodology are provided in the previous article (51).

In the study moonlighting was defined as additional paid work – whether of a nursing or non-nursing nature – done by nurses in a private health facility, another government health facility, an insurance company, private health laboratory, or in the same health care facility while holding a primary, paid nursing job, but excluding overtime (51). Intention to leave was measured through one question: ‘In the next 12 months, do you plan to leave your current primary job?’

Data were weighted to reflect the population distribution of nurses between the public and private health sectors, and the four study provinces, and analysed using STATA^®^ 13. We also adjusted for the clustering and stratification introduced by the sampling design. Frequency tabulations were done to describe the socio-demographic characteristics of the respondents. Cross-tabulations were done to investigate associations of each of the factors, including moonlighting, with the intention to leave employment in the 12 months following the survey, our main outcome of interest. Bivariate logistic regression models were fitted and only factors found to be statistically significantly associated with intention to leave at a conservative 20% level were considered in the final model-building process using a multiple logistic regression model. All other statistical tests were carried out at 5% significance level.

## Results

### Participant characteristics

The unweighted demographic and background characteristics of the 3,784 nurses recruited in the four study provinces are shown in Table 1. The majority of survey participants were female (92.7%), and employed in provincial government (52.8%). The participants were predominantly middle-aged, with a mean age of 41.5 (SD±10.4) years. A few respondents omitted to complete some of the questions which accounts for the minor variations in denominators.

**Table 1:** Demographic and employment characteristics of survey participants

Characteristic	Total (*n*=3,784)
Mean age (standard deviation)	41.5 (10.4)
Age group (years)	
< 25	150 (4.1%)
25–34	888 (24.1%)
35–44	1,112 (30.2%)
45–54	1,115 (30.3%)
55+	414 (11.2%)
Sex	
Female	3,489 (92.7%)
Male	276 (7.3%)
Marital status	
Married	1,693 (45.0%)
Living together	130 (3.5%)
Single	1,328 (35.3%)
Divorced/separated	410 (10.9%)
Widowed	201 (5.3%)
Children	
Median number of children (range)	2 (1–14)
Median age of youngest child	12
Nursing category	
Professional nurse	1,910 (51.5%)
Enrolled nurse	818 (22.1%)
Auxiliary nurse	982 (26.5%)
Median years qualified as a nurse (mean)	15 (15.9)
Primary job (sector)	
Provincial government	1,955 (52.8%)
Private sector	1,400 (37.8%)
Nursing agency	346 (9.4%)
Median years at primary job (mean; range)	7 (10.3; 1–47)
Unit of work	
Paediatric critical care	183 (5.1%)
Adult critical care	421 (11.8%)
High care	33 (0.9%)
Theatre	668 (18.8%)
Emergency	392 (11.0%)
Maternity	574 (16.1%)
General wards	1,140 (32.0%)
Psychiatry	117 (3.3%)
Outpatient department	30 (0.8%)

### Factors influencing nurses’ intention to leave

In the study, 1,086 participants (30.9%) indicated that they planned to leave their primary jobs in the 12 months following the survey. Of these, 15.4% indicated that they planned to go overseas, 36.7% to move to another public sector job, and 13.5% to another private sector job. The remainder were made up of smaller proportions planning to work in nursing agencies or non-governmental organisations, or who planned to study, retire, or stay at home in the following year.

The study participants’ intention to leave their primary jobs varied by age, province, years at the primary job, nursing category, number of children, and moonlighting status (Table 2). 37.7% of nurses aged 25–34 years indicated their intention to leave, followed by 32.6% of nurses aged 35–44 years. Intention to leave was higher among nurses with no children (37.8%), compared to those with one child (30.4%), two children (30.9%), or four or more children (24.4%). In Gauteng Province, 37.8% of nurses indicated their intention to leave, compared to the Free State (28.1%), Western Cape (25.9%), or Eastern Cape (25.5%). Intention to leave was also higher among nurses working for a commercial nursing agency (44.1%) or the private health sector (36.9%), compared to the provincial government (28.2%). Those nurses with less than 1 year (22.3%) or 15–19 years of service (29.4%) were less likely to intend leaving their primary job (*p*<0.001) compared to those with between 5 and 9 years of service (39.2%).

**Table 2:** Bivariate analysis of factors influencing nurses’ intention to leave their jobs within 12 months

Variable	*n*	Intention to leave (%)	*P*
Total	3,513	1,086 (30.9%)	
Moonlighting in the past 12 months			<0.001
No	2,477	690 (27.9%)	
Yes	965	381 (39.5%)	
Province			<0.001
Gauteng	1,461	552 (37.8%)	
Eastern Cape	935	239 (25.5%)	
Western Cape	795	206 (25.9%)	
Free State	322	91 (28.1%)	
Age group (in years)			<0.001
< 25	126	34 (26.8%)	
25–34	864	326 (37.7%)	
35–44	981	320 (32.6%)	
45–54	1,026	265 (25.8%)	
55 +	410	106 (25.8%)	
Sex			0.181
Male	272	96 (35.3%)	
Female	3,234	988 (30.6%)	
Marital status			0.447
Married/living together	1,592	495 (31.1%)	
Single	1,406	447 (31.8%)	
Divorced/widowed	503	142 (28.2%)	
Number of children			0.005
None	593	224 (37.8%)	
One	794	241 (30.4%)	
Two	1,084	335 (30.9%)	
Three	669	194 (29.0%)	
Four or more	363	88 (24.4%)	
Sector			<0.001
Public	2,561	722 (28.2%)	
Private	671	248 (36.9%)	
Agency	216	95 (44.1%)	
Nursing category			<0.001
Professional nurse	1,682	609 (36.2%)	
Enrolled nurse	699	227 (32.5%)	
Nursing assistant	1,132	251 (22.1%)	
Years working at primary job			<0.001
Less than 1	379	84 (22.3%)	
1–4	911	321 (35.2%)	
5–9	652	255 (39.2%)	
10–14	323	100 (31.1%)	
15–19	298	88 (29.4%)	
20 or more	873	219 (25.1%)	

Table 2 also shows that intention to leave varied by nursing category (*p*<0.001). It was highest among professional or registered nurses with 4 years of training (36.2%), than among enrolled nurses with 2 years of training (32.5%) or among nursing assistants with 1 year of training (22.1%). Lastly, the proportion of participants with intention to leave in the 12 months following the survey was higher among the moonlighters compared to non-moonlighters (39.5% vs. 27.9%; *p*<0.001). Among those planning to go overseas, 18% (82) of moonlighters, compared to 13.8% (89) of non-moonlighters planned to go overseas, but this difference was not statistically significant (*p*=0.06).

### Predictors of intention to leave

In the multiple logistic regression analysis, predictors of intention to leave were: moonlighting in the preceding year, nursing category, sector of primary employment, period working at the primary job, and number of children (Table 3).

**Table 3:** Final multiple logistic regression model results for factors associated with nurses’ intention to leave their primary jobs within 12 months

Variable	Odds ratio	[95% CI]	*P*
Moonlighting in the previous 12 months			
No	–		–
Yes	1.40	[1.16–1.69]	<0.001
Province			
Gauteng	–	–	–
Eastern Cape	0.70	[0.54–0.91]	0.008
Western Cape	0.60	[0.49–0.75]	<0.001
Free State	0.71	[0.56–0.89]	0.004
Sector			
Provincial government	–	–	–
Private sector	1.11	[0.91–1.35]	0.293
Nursing agency	2.09	[1.49–2.94]	<0.001
Years working at primary job			
<1	–	–	–
1–4	2.21	[1.59–3.07]	<0.001
5–9	2.55	[1.80–3.61]	<0.001
10–14	1.69	[1.13–2.53]	0.011
15–19	1.70	[1.11–2.61]	0.016
20 or more	1.61	[1.12–2.31]	0.010
Nursing category			
Professional nurse	–	–	–
Enrolled nurse	0.79	[0.62–1.01]	0.058
Nursing assistant	0.53	[0.42–0.66]	<0.001
Number of children			
None	–	–	–
One	0.71	[0.54–0.95]	0.019
Two	0.71	[0.55–0.92]	0.010
Three	0.70	[0.52–0.95]	0.022
Four or more	0.59	[0.40–0.87]	0.008
Constant	0.44	[0.31–0.64]	<0.001

The weighted crude (unadjusted) odds for intention to leave the primary job in the 12 months following the survey were 1.69 (95% CI: 1.41–2.02) times higher among the moonlighters compared to the non-moonlighters. This was still significant (OR=1.40, 95% CI: 1.16–1.69) after adjusting for other factors such as nursing category, sector of primary job and years working in primary job, sector and province of primary employment.

The adjusted analysis shows that individuals working for a commercial nursing agency (OR=2.09, 95% CI: 1.49–2.94) were more likely to express intention to leave, compared to those working in the provincial government. Enrolled nurses (OR=0.79, 95% CI: 0.62–1.01) or nursing assistants (OR=0.53, 95% CI: 0.42–0.66) were less likely to report intention to leave compared to professional nurses. The odds of individuals who have worked for 1–4 years to report intention to leave their primary job in the 12 months following the survey were 2.21 (92% CI: 1.59–3.07) times higher compared to those who have worked for less than 1 year. The intentions peak among those with 5–9 years’ experience (OR=2.55, 95% CI: 1.80–3.61). In later years of working experience, although the odds of participants reporting intention (ORs: 1.61–1.70) to leave were higher, the results show a fall in such intentions in relation to the reference group. The adjusted results also show a decline in nurses’ intentions to leave their primary jobs with increasing number of children. The odds ratios range from 0.71 (95% CI: 0.54–0.95) among those with one child to 0.59 (95% CI: 0.40–0.87) among those with four or more children compared to those with no children.

## Discussion

We found that almost one-third (30.9%) of respondents indicated their intention to leave their primary employment in the 12 months following the survey. This figure was lower than the turnover intent of nurses found in other studies done in South Africa in recent years (19, 26). A 2005 study to examine the relationship between job satisfaction, turnover intent, and demographic variables among primary health care nurses in a rural South African area found that 51.1% of these nurses considered leaving within 2 years following the study (19). Another study that aimed to compare the job satisfaction and intention to leave among different categories of health workers in Tanzania, Malawi, and South Africa found that 41.4% of South African health workers indicated that they were actively seeking other jobs, compared to 26.5% in Malawi, and 18.1% in Tanzania (26). However, these studies are not directly comparable as they comprised of different study populations, with different approaches to the measurement of intention to leave.

There are wide variations in study findings on turnover intent. The finding of 30.9% turnover intent in our study is similar to that found in Belgian hospitals done as part of a multi-country study – 30% of Belgian registered nurses indicated that they planned to leave their jobs (52). The multi-country hospital study, done in Europe and the United States of America (USA), examined patient safety, satisfaction, quality of hospital care, and nurse outcomes and found that intention to leave ranged from a low of 14% in the USA to a high of 49% in Greece and Finland (52). A 2007 study in Senegal among public sector midwives found that 58.9% of midwives reported their intention to leave within a year (28). This is in contrast to a study in Chinese hospitals that found that only 5% of nurses indicated their intention to leave (29). These differences in turnover intent might be explained by different contexts, study populations, and measurement methods.

In the multiple logistic regression analysis, the predictors of intention to leave were: category of nurse, primary employment in a commercial nursing agency, working for between 1 and 10 years at the primary job, and moonlighting in the preceding year (Table 3).

Enrolled nurses or nursing assistants were less likely to report intention to leave compared to professional nurses (Table 3). This may reflect the higher qualifications and skills of professional (registered) nurses, with greater potential for international movement and transferability of experience and skills. We could not find many studies that examine the relationship between nursing category, and intention to leave, as the bulk of the literature focuses on registered (professional) nurses (4, 13, 15, 42). A United Kingdom study that focused primarily on appropriate retention strategies found that enrolled nurses had greater job satisfaction than registered nurses and lower intention to quit, which may have been the result of ‘lower expectations in terms of pay and promotion due to their constrained promotion prospects’ (30, p. 689).

Surprisingly, nurses working for a commercial nursing agency were more likely to indicate their intention to leave, compared to those working in the public health sector. This may be related to the timing of the study, as there was a major public sector financial incentive policy implemented 2 years prior to the survey, which assisted in attracting large numbers of nurses back to the public health sector (53, 54).

The study found that those nurses working for between 1 and 10 years at the primary job were more likely to indicate their intention to leave. A 2005 study to examine the relationship between job satisfaction, turnover intent, and demographic variables among primary health care nurses in a rural area of South Africa found that turnover intent was significantly and inversely correlated with the number of years of nursing (19). However, the literature suggests that the relationship between length of service (tenure) and turnover is complex, because of possible confounding factors such as context, work environment, experience, and age (4, 13, 17, 25, 29, 42, 43, 45).

This is one of the first studies to examine the relationship between moonlighting and nurses’ intention to leave. We found a significant association between moonlighting and nurses’ intention to leave their primary jobs (39.5% among the moonlighters compared to 28% among non-moonlighters). The adjusted odds for intention to leave the primary job in the 12 months following the survey were 1.40 times higher among moonlighting nurses compared to the non-moonlighters. Organisational development researchers have suggested that moonlighting provides workers with an alternative source of income, training, and benefits, thus influencing turnover (47). This theory was supported by the 2010 moonlighting survey in South Africa that found multiple and varied motivations for moonlighting, including financial reasons and non-financial reasons such as taking care of patients, the opportunity to learn new nursing skills, and collegial relationships (51). However, moonlighting could also change staff perceptions, decisions, and behaviours, which may impact on turnover at their primary jobs either positively (in the decision to stay because of alternative benefits provided by the secondary job) or negatively (in accelerating actual turnover) (47). Our study suggests that moonlighting may accelerate nurse turnover intent. Although other studies have demonstrated that nurse absenteeism predicts turnover (11, 48, 55), we could not find similar or comparable studies on moonlighting and intention to leave in other low- or middle-income country settings, or even in high-income countries. Hence, more research is needed to determine whether and how moonlighting contributes to high nurse turnover intent, and ultimately to turnover.

The limitations of the study include: the cross-sectional study design which can only capture nurses remaining in their jobs; the possible social desirability bias resulting in lower disclosures of dissatisfaction, moonlighting, or intention to leave; and the fact that nurses who had not formally obtained permission for moonlighting may have been reluctant to admit to it. These limitations are discussed in more detail in the previous article (51). Although intention to leave is a very strong predictor of actual turnover, the expressed intentions of these nurses may not result in actual turnover. For example, the midwives study in Senegal found that although 58.9% reported their intention to leave within a year, the annual turnover rate was found to be only 9% due to limited job alternatives (28). Notwithstanding these limitations, our study is one of the first to examine the relationship between moonlighting and intention to leave. The study also enhances our understanding of the under-explored concept of moonlighting among South African nurses, who may be using moonlighting as a way of finding out whether changing jobs is possible and facilitating their decision-making to leave.

Our study findings have implications for health workforce policies and management, and for quality of care. As indicated above, high nurse turnover has the potential to affect health care provision to patients (3, 11, 13, 30, 31) and the morale, performance, and productivity of the remaining nurses (13, 32).

South Africa's five-year plan on human resources for health emphasises the importance of staff retention, both as a strategic imperative and as an outcome (49). Similarly, the strategic plan on nurse education, training, and practice highlights the importance of nurse retention (56). Moonlighting and high nurse turnover have to be addressed in tandem, as these issues have significant implications for quality of care in health facilities. The importance of safe, quality care to patients in South Africa is emphasised by the national core standards (57).

Although both staff retention and mechanisms to address quality of care are highlighted in various policy documents (49, 56, 57), a key challenge in South Africa has been in translating laudable plans and strategies into action (58, 59). Much more concrete action is needed to create positive practice environments, as there is evidence that these improve nurse retention and quality of patient care (13, 60, 61). In practical terms, strategies to create positive practice environments include: nurse participation in organisational matters; nursing practice which is flexible, meaningful and effective; leadership and support by nurse managers; adequate staffing and resources; and collaborative doctor-nurse relationships (61, p. 88).

A first step in the management of moonlighting is to recognise that it is widespread (51) and motivated by both financial and non-financial reasons. Hence, there is need for dialogue and debate on moonlighting in the South African health system, and the ethical and accountability issues that arise from nurses engaging in a second job, while employed full-time. The non-financial reasons for moonlighting such as recognition and appreciation for exemplary nursing services link to the strategies needed to create positive practice environments (60, 61). Key strategies such as participatory workplace forums and enhanced teamwork and collegial relationships do not require significant additional money or resources.

In the medium term, a uniform national monitoring and evaluation system should be developed, which includes indicators such as nurses’ absenteeism rates and trends, and total number of hours worked by each nurse, through improved employer personnel or nursing council information systems.

## Conclusions

Both the public and private health sectors in South Africa have a statutory duty to provide the best possible health care to patients within available resources and to achieve a balance between the rights and duties of health care providers (62). The study has found an association between moonlighting and intention to leave, which would need to be confirmed by other studies. The study points to the need for improved management of moonlighting and implementation of strategies for nurse retention in the South African health system.
